# MTHFD2 promotes PD‐L1 expression via activation of the JAK/STAT signalling pathway in bladder cancer

**DOI:** 10.1111/jcmm.17863

**Published:** 2023-07-21

**Authors:** Linzhi Li, Yunlong Zhang, Weimin Hu, Fan Zou, Jinzhuo Ning, Ting Rao, Yuan Ruan, Weimin Yu, Fan Cheng

**Affiliations:** ^1^ Department of Urology Renmin Hospital of Wuhan University Wuhan China

**Keywords:** bladder cancer (BC), malignant phenotype, MTHFD2, PD‐L1, poor prognosis

## Abstract

Although combination chemotherapy is widely used for bladder cancer (BC) treatment, the recurrence and progression rates remain high. Therefore, novel therapeutic targets are required. Methylenetetrahydrofolate dehydrogenase 2 (MTHFD2) contributes to tumourigenesis and immune evasion in several cancers; however, its biological function in BC remains unknown. This study aimed to investigate the expression, prognostic value and protumoural function of MTHFD2 in BC and elucidate the mechanism of programmed death‐ligand 1 (PD‐L1) upregulation by MTHFD2. An analysis using publicly available databases revealed that a high MTHFD2 expression was correlated with clinical features and a poor prognosis in BC. Furthermore, MTHFD2 promoted the growth, migration, invasion and tumourigenicity and decreased the apoptosis of BC cells in vivo and in vitro. The results obtained from databases showed that MTHFD2 expression was correlated with immune infiltration levels, PD‐L1 expression, and the Janus kinase/signal transducer and activator of transcription (JAK/STAT) pathway. The expression of MTHFD2, PD‐L1 and JAK/STAT signalling pathway‐related proteins increased after interferon gamma treatment and decreased after MTHFD2 knockdown. Moreover, addition of a JAK/STAT pathway activator partially reduced the effect of MTHFD2 knockdown on BC cells. Collectively, our findings suggest that MTHFD2 promotes the expression of PD‐L1 through the JAK/STAT signalling pathway in BC.

## 1 INTRODUCTION

Bladder cancer (BC) is the 10th most common malignancy worldwide and the most common cancer of the urinary system, with 57,278 new cases and 212,536 bladder cancer‐related deaths estimated in 2020.^1^ Currently, immune checkpoint inhibitors (ICIs) are approved by the US Food and Drug Administration for the treatment of patients with BC.^2^ However, only a subset of patients achieve long‐term durable responses. Therefore, new treatment options are needed.

Methylenetetrahydrofolate dehydrogenase 2 (MTHFD2), which has bifunctional dehydrogenase and cyclohydrolase activities, is a key enzyme involved in mitochondrial one‐carbon folate metabolism.^3^ MTHFD2 is overexpressed in a variety of tumours but exhibits low or no expression in most differentiated adult tissues.^4^ Accumulating evidence indicates that MTHFD2 is related to tumourigenesis, tumour development, poor disease outcomes and drug resistance.^5^ Moreover, recent findings have uncovered a nonmetabolic role of MTHFD2 in tumour immune evasion.^6^ Taken together, these studies suggest that MTHFD2 is oncogenic and may be an important prognostic indicator as well as a good immunotherapy target in cancers. Programmed death‐ligand 1 (PD‐L1), a ligand for the immune checkpoint receptor programmed death 1 (PD‐1) is frequently expressed in various tumour cells.^7^, ^8^ Binding of PD‐L1 to PD‐1 on activated T cells inhibits antitumor immunity by counteracting T cell‐activating signals.^9^ In BC, a high PD‐L1 expression is associated with poor survival outcomes in patients with advanced and aggressive tumours.^10^ However, single‐agent PD‐L1 therapy is not as effective as expected. The response to PD‐L1 inhibitor treatment is correlated with PD‐L1 expression levels.^2^ Therefore, regulation of PD‐L1 levels in BC should be explored. Additionally, novel immuno‐targeted combination therapies for advanced BC need to be developed.

The Janus kinase/signal transducer and activator of transcription (JAK/STAT) signalling pathway has been reported to arbitrate the transcription pathways of a variety of cytokines in various human cancers, including BC, resulting in the regulation of cell proliferation, migration, invasion, apoptosis, survival, immune response and other biological processes.^11^, ^12^, ^13^ Notably, activation of the JAK/STAT pathway can drive PD‐L1 expression.^14^ Therefore, we hypothesized that MTHFD2 is involved in regulating PD‐L1 expression in BC via the JAK/STAT signalling pathway.

This study aimed to examine the expression, prognostic value and protumoural function of MTHFD2 in BC and further investigate the mechanism of PD‐L1 upregulation by MTHFD2.

## 2 MATERIALS AND METHODS

### 2.1 Data mining and bioinformatics analyses

The different expression of MTHFD2 in cancers was analysed by the tumour immune estimation resource (TIMER) online system (https://cistrome.shinyapps.io/timer/). TIMER was also used for a comprehensive analysis of tumour‐infiltrating immune cells and the correlation between MTHFD2 expression and common immune checkpoint molecules. The MTHFD2 expression RNA sequencing (RNA‐seq) data and corresponding clinical data were downloaded from bladder cancer projects (TCGA‐BLCA) level 3 HTSeq‐Fragments Per Kilobase per Million (FPKM) in The Cancer Genome Atlas (TCGA) official website (https://portal.gdc.cancer.gov/). FPKM data were transformed using log2 for further calculation. R 4.2.1 was applied for all data processing and statistical analysis. Kaplan–Meier survival analysis was performed using the Gene Expression Profiling Interactive Analysis (GEPIA) (http://gepia.cancer‐pku.cn/), a web‐based tool containing high‐throughput RNA sequencing data (TCGA and GTEx databases). To explore the biological processes enriched by MTHFD2 in BC, GO/KEGG term enrichment analyses were performed using R packages. We additionally performed gene set enrichment analysis (GSEA) using the C2.cp.kegg.v2022.1.Hs.symbols.gmt with GSEA 4.3.0 software. The samples were divided into a high MTHFD2 expression group (205 samples) and a low MTHFD2 expression group (206 samples) in TCGA database. Gene sets with nominal *p*‐value < 0.05 and false discovery rate (FDR) < 0.25 were considered of statistical significance.

### 2.2 Immunohistochemical staining

The fresh tumour tissues and adjacent nontumour tissues of eight patients with BC were obtained from Renmin Hospital of Wuhan University. This study has been approved by the Ethics Committee of Medical School of Wuhan University. All resected tissues were fixed with 4% paraformaldehyde and embedded in paraffin. Then, paraffin‐embedded tissues were sectioned into 5 μm thick. The tissue sections were dewaxed and the endogenous peroxidase was blocked by 1% hydrogen peroxide. Next, the sections were incubated with primary antibodies MTHFD2 (1:200, PHF6156, Abmart), p‐JAK1 (1:200, TP56310, Abmart), p‐STAT3 (1:100, T56566, Abmart) and PD‐L1 (1:50, T55980, Abmart) overnight at 4°C in a humidified box. After being washed, the sections were further incubated with a secondary anti‐rabbit antibody for 1 h at room temperature. Finally, the sections were reacted with 3,3‐diaminobenzidine (DAB) and counterstained with haematoxylin. The histochemistry score (H‐score) of protein expression was calculated by multiplying the staining intensity (0, negative; 1, weak; 2, moderate; and 3, strong) and the percentage of positive cells (0, 0%–10%; 1, 11%–25%; 2, 26%–50%; 3, 51%–75%; and 4, >75%).

### 2.3 Cell culture

The normal human bladder uroepithelium cell line SV‐HUC‐1 and human BC cell lines (5637, T24, UMUC3) were obtained from the Chinese Academy of Sciences. SV‐HUC‐1 were cultured in the F12K medium (Pricella). T24 and 5637 were cultured in RPMI‐1640 medium (Pricella). UMUC3 were cultured in DMEM (Pricella). All culture media contained 10% foetal bovine serum (Pricella) and 1% penicillin/streptomycin (Pricella). All cells were cultured in a humidified incubator at 37°C and 5% CO2.

### 2.4 Lentiviral transfection and reagent treatment

To construct MTHFD2 stable knockdown T24 and 5637 BC cell lines, lentivirus shRNAs were synthesized by OBiO Co., Ltd. T24 and 5637 cells were incubated with MTHFD2 shRNAs and non‐silencing control (NC). After transfection, cells were given 24 h to recover before selection in 4 μg/mL puromycin (Beyotime) for 2–4 days. The knockdown efficiency was validated by Real‐Time Quantitative PCR and western blotting. And phosphate buffered saline (PBS)‐treated cells were included as the control group. T24 and 5637 cells were divided into the control group, the lentivirus‐mediated shNC group and the shMTHFD2 group for further experiments.

For interferon gamma (IFN‐γ) stimulation, cells were grown to approximately 80% confluence and treated with 20 ng/mL recombinant human IFN‐γ (MedChemExpress) for 24 h.

For activation of JAK/STAT pathway, cells were incubated in RPMI‐1640 medium with 20 μM of JAK/STAT pathway activator RO8191^15^, ^16^ (MedChemExpress).

### 2.5 Quantitative reverse transcription polymerase chain reaction (RT‐qPCR)

Total RNA from cells was extracted using TRIzol reagent (Servicebio) and reversely transcripted to cDNA using the SweScript All‐in‐One First‐Strand cDNA Synthesis SuperMix for qPCR (Servicebio). RT‐qPCR was performed with 2 × SYBR Green qPCR Master Mix (Servicebio) according to the manufacturer's instructions. The relative expression of genes was calculated using ${{2^{-}}^{\Delta \Delta }}^{C_{t}}$ method^17^ and normalized to the expression of GAPDH. The RT‐qPCR primers were as follows:

GAPDH forward, 5′‐GGAAGCTTGTCATCAATGGAAATC‐3′;
GAPDH reverse, 5′‐TGATGACCCTTTTGGCTCCC‐3′;
MTHFD2 forward, 5′‐TCCTGGTTGGCGAGAATCCT‐3′;
MTHFD2 reverse, 5′‐CCCCATGGAGTAGCCGGTAA‐3′.

### 2.6 Cell viability assays

Cell viability was measured using the Cell Counting Kit‐8 (CCK‐8; Servicebio). Cells were digested and then seeded into a 96‐well plate at a density of 1000 cells/well. When cultured to 0, 24, 48, 72 and 96 h, cells were treated with CCK‐8 (10 μL/well) and incubated at 37°C for 1 h. We record the absorbance at 450 nm using a microplate reader.

The colony formation assay was also performed to test cell viability. Cells were digested and then seeded into a 6‐well plate at a density of 500 cells/well. After being cultured for 2 weeks, cells were fixed with 4% paraformaldehyde and stained with gentian violet. And pictures were taken using a digital camera.

### 2.7 Wound healing assay

Cell migratory ability was evaluated by wound healing assay. Cells were seeded into 6‐well plates and were cultured to confluency. The wounds were created by scraping the cell layers using 200 μL plastic pipette tips. The wounds were observed immediately after the scratch, and again 36 h later. Images were captured and the gap distances of migrating cells were measured using a microscope (OLYMPUS IX71).

### 2.8 Transwell cell invasion assay

For the invasion assay, cells were digested and resuspended in serum‐free medium. Then cells in 200 μL serum‐free medium were seeded into the upper chamber at a density of 8 × 10^4^ cells/well, and the upper chamber was coated with Matrigel (BD Biosciences). 600 μL of RPMI‐1640 medium containing 10% FBS was added to the lower chamber. After being cultured for 72 h, cells were fixed with 4% paraformaldehyde and stained with gentian violet. Images of the invaded cells were acquired by a microscope (Olympus IX71) and cells were counted in three randomly selected fields.

### 2.9 Apoptosis and cell cycle analysis

Apoptosis and cell cycle analysis were assessed by flow cytometry according to standard protocols. Cells were seeded into 6‐well plates and were harvested at a density of 1 × 10^6^ cells/well. After being washed twice in PBS, cell apoptosis was measured by flow cytometry (CytoFLEX, Beckman Coulter) using the Annexin V‐FITC/PI apoptosis detection kit (YEASEN). And cell cycle analysis was measured with the propidium iodide (YEASEN). Data were analysed with FlowJo 10.6.2 software.

### 2.10 Detection of mitochondrial membrane potential (MMP)

MMP in T24 and 5637 cells was detected by Mitochondrial Membrane Potential assay kit with JC‐1 (Beyotime) in accordance with the manufacturer's guidelines. Briefly, cells were harvested and stained with JC‐1 (1×) for 20 min at 37°C. Then, the micrographs were recorded under a fluorescent inverted microscope (OLYMPUS IX71) at 100× magnification, and the red JC‐1 fluorescence intensity was quantified by ImageJ.

### 2.11 Immunofluorescence staining

Cells were seeded on slides in 6‐well plates and were cultured for 24 h. Then, cells were fixed with 4% paraformaldehyde and permeabilised by TritonX‐100 for 20 min at room temperature. Following washes, cells were blocked with BSA for 30 min and then incubated with primary antibodies overnight at 4°C in a humidified box. The primary antibodies were as follows: Ki67 (1:200, 27309‐1‐AP, Proteintech); Vimentin (1:50, 10366‐1‐AP, Proteintech); PD‐L1 (1:500, ab205921, Abcam). After rinsing, cells were further incubated with fluorescently labelled secondary antibodies for 1 h in the dark. Finally, nuclei were counterstained with DAPI. The fluorescence images were captured by a fluorescent inverted microscope (OLYMPUS IX71).

### 2.12 Western blot (WB) analysis

Total cellular proteins were extracted with RIPA lysis buffer (Servicebio) and measured using the BCA protein quantification kit (Beyotime). Equivalent amounts of protein samples were loaded in each lane and separated by 10% sodium dodecyl sulphate‐polyacrylamide gel electrophoresis (SDS‐PAGE). Subsequently, the samples were transferred to polyvinylidene fluoride (PVDF) membranes. After blocking with 5% skim milk, the membranes were incubated with a primary antibody overnight at 4°C. The primary antibodies were as follows: MTHFD2 (1:5000, PHF6156, Abmart); PD‐L1 (1:500, T55980, Abmart); p‐JAK1 (1:2000, TP56310, Abmart); JAK1 (1:2000, T57173, Abmart); p‐STAT3 (1:1000, T56566, Abmart); STAT3 (1:2000, T55292, Abmart); Bcl‐2 (1:500, WL01556, Wanleibio); Bax (1:1000, WL01637, Wanleibio); Cyclin D1 (1:5000, 60186‐1‐Ig, Proteintech) and β‐Tubulin (1:5000, M20005, Abmart). Endogenous β‐tubulin was performed as an internal control protein for WB analysis. After washing with tris‐buffered saline with 0.1% Tween‐20 (TBST), the membranes were incubated with secondary antibodies for 1 h at room temperature. All bands were measured using an enhanced chemiluminescence (ECL) system kit (Servicebio) and analysed using ImageJ software. The statistical graphs about WB was presented in Figure S1.

### 2.13 Nude mouse xenograft assay

Four‐week‐old nude mice were purchased from the Centre of Experimental Animals at Wuhan University Medicine College (Hubei, China). All nude mice were kept in a standard temperature‐controlled isolation package and allowed to drink and eat freely. For the nude mouse xenograft assay, T24 cells (2 × 10^6^) resuspended in 100 μL of PBS were injected subcutaneously into each nude mouse. The growth of tumour size was evaluated weekly post‐injection with a vernier calliper, and tumour volumes were calculated as follows: V = L × W × W/2. After 28 days, mice were euthanized and the tumours were subsequently harvested for further research.

### 2.14 TUNEL Detection

The In Situ Apoptosis Detection kit (Roche Applied Science) was used to perform the TUNEL assay according to the manufacturer's protocol. Briefly, the tissue samples were prepared, and apoptotic cells were labelled by terminal transferase‐medicated dUTP nick‐end labelling. Finally, the images were acquired by a fluorescent inverted microscope (OLYMPUS IX71).

### 2.15 Statistical analysis

GraphPad Prism 8 software was used for all statistical analyses and data visualisation. The Shapiro–Wilk test was used to assess normality. For the data with normal distribution, Student's *t*‐test was used to analyse differences between two independent groups. And one‐way analysis of variance (anova) followed by Bonferroni's test was used to analyse differences among multiple groups. For the data with non‐normal distribution, we performed the Mann–Whitney U test to analyse differences between two groups and the Kruskal–Wallis H test, followed by Dunn's test to analyse differences among multiple groups. The Spearman rank correlation test was used to analyse the relationship among the expressions of several proteins. All data were represented as means ± standard deviations (SD). A *p*‐value < 0.05 was considered of statistical significance.

## 3 RESULTS

### 3.1 Upregulation of MTHFD2 in various cancer types including BC

**FIGURE 1:**
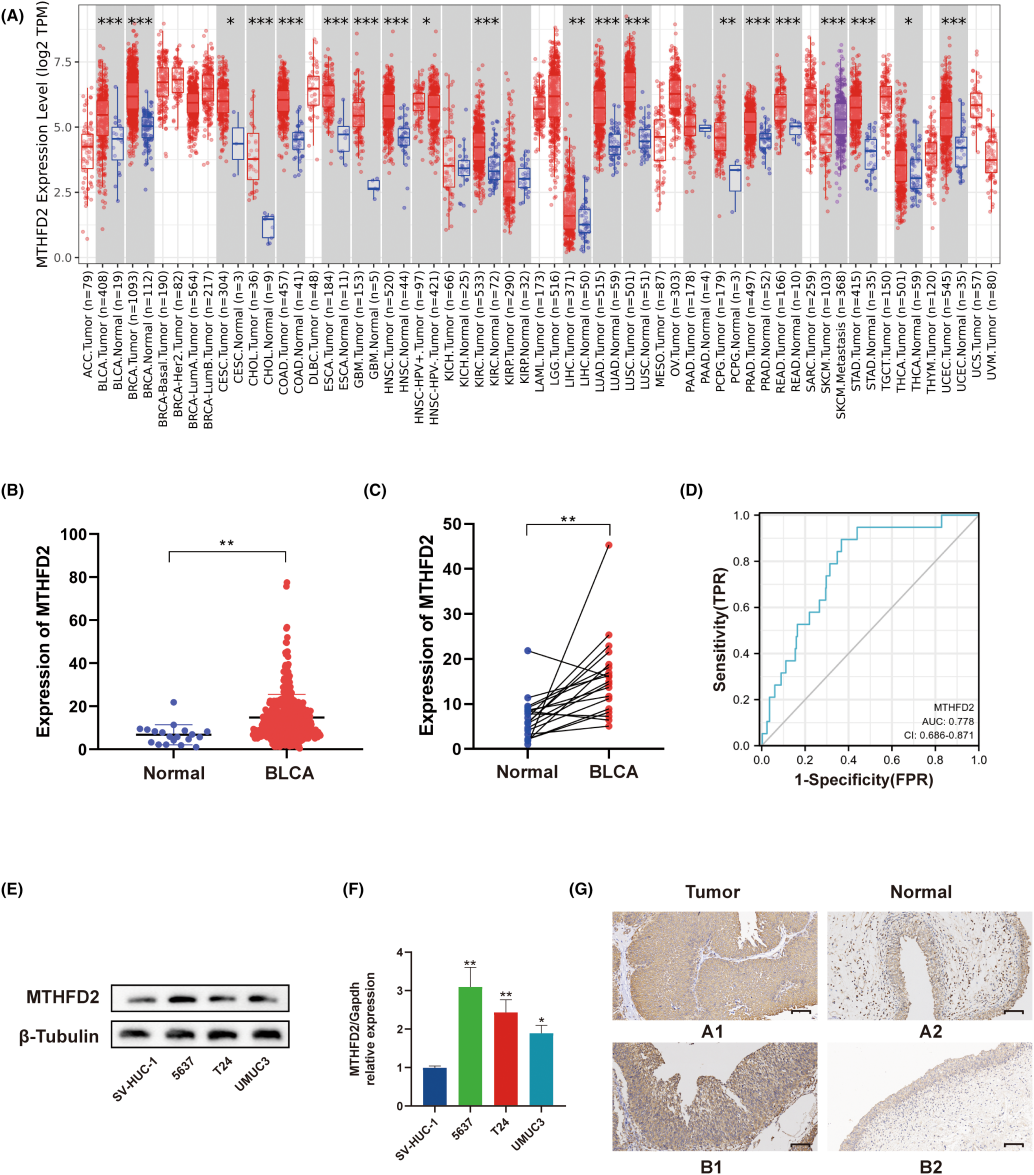
MTHFD2 expression in various cancers notably in BC (A) Differential expression of MTHFD2 in various types of cancer analysed by TIMER. (B) Differential expression of MTHFD2 in BC tissue samples and normal bladder tissue samples. (C) Differential expression of MTHFD2 in BC tissue samples and their paired normal bladder tissue samples. (D) ROC curve analysis of MTHFD2. (E) WB analysis of MTHFD2 expression in SV‐HUC‐1 and bladder cancer cell lines. (F) RT‐qPCR analysis of MTHFD2 expression in SV‐HUC‐1 and bladder cancer cell lines. (G) Representative images of immunohistochemistry for the tumour and adjacent normal tissues of eight BC patients. MTHFD2 was used for staining. (Scale bar represents 100 μm) (*p < 0.05; **p < 0.01; ***p < 0.001).

To assess the MTHFD2 expression in cancers, we analysed the TIMER database and verified the results using the GEPIA. We found MTFHD2 was significantly upregulated in multiple types of cancer, including bladder cancer, breast cancer, renal clear cell carcinoma and prostate cancer (Figure 1A; Figure S2). To further explore the MTHFD2 expression in BC, we analysed RNA‐seq data from BLCA project in TCGA. As shown in Figure 1B, MTHFD2 was overexpressed in BC samples compared with normal tissue samples. The same results were seen in the comparison between BC samples and their paired normal tissue samples (Figure 1C). Then we explored its diagnostic value for BC patients. The results of ROC curves revealed that the AUC was 0.778 (0.686–0.871, 95% CI, *p* < 0.01) in a comparison between BC samples and matched normal samples (Figure 1D).

To make a strong conclusion, we performed WB analysis and RT‐qPCR to detect the protein and mRNA levels of MTHFD2 in cell lines. As expected, MTHFD2 was overexpressed in three BC cell lines (5637, T24, UMUC3) compared to normal human bladder uroepithelium cell line (SV‐HUC‐1) (Figure 1E,F). Furthermore, we undertook immunohistochemistry staining for MTHFD2 in the tumour and adjacent normal tissues of eight patients with BC. The results showed that MTHFD2 had higher expression in tumour tissues than peritumour tissues (Figure 1G, the H‐score of the tumour and adjacent normal tissues of eight BC patients was presented in Figure S3).

### 3.2 MTHFD2 was associated with the clinicopathologic features and prognosis of BC

**FIGURE 2:**
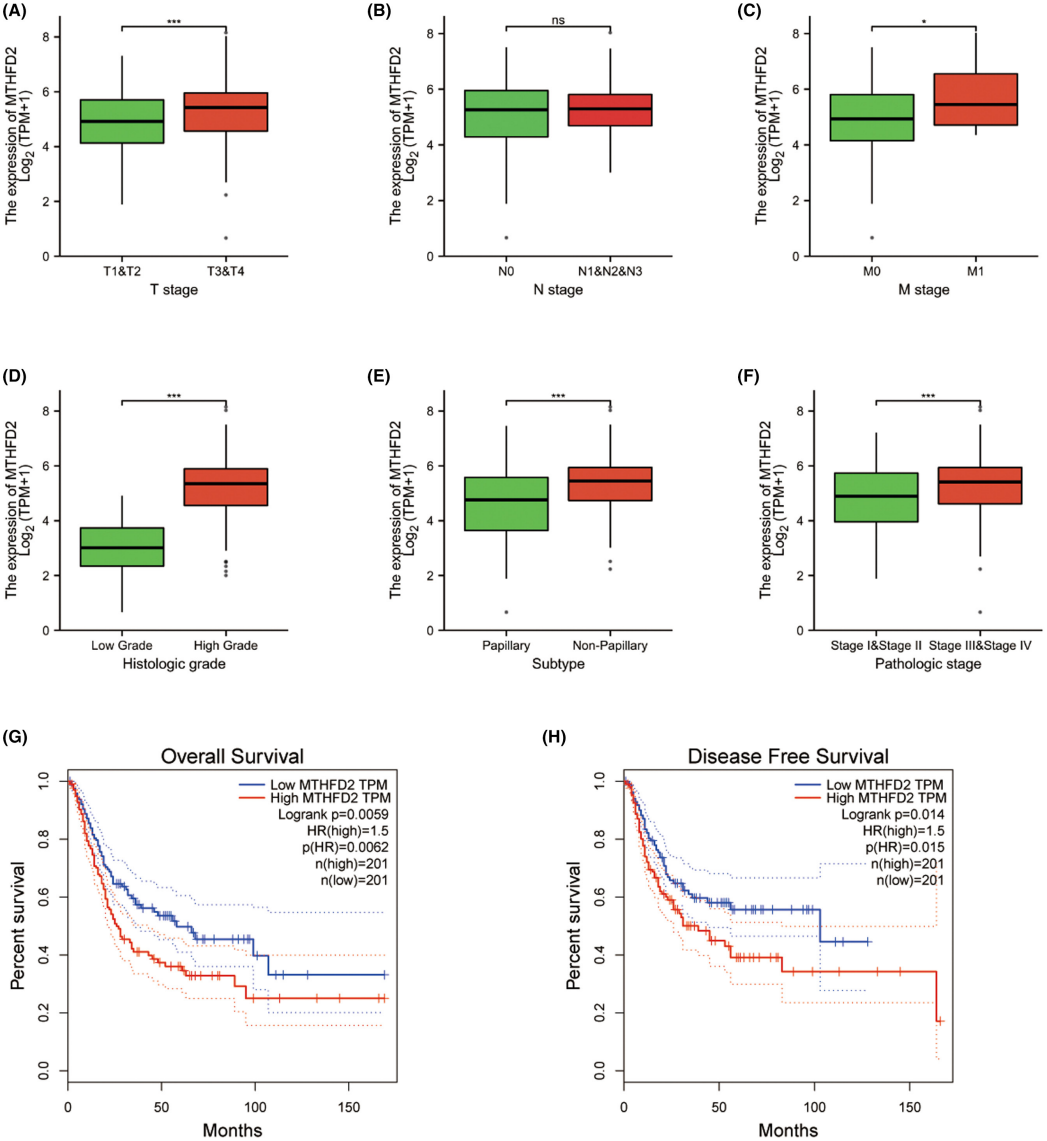
Clinical correlation of MTHFD2 (A–F) Expression of the MTHFD2 gene among distinct clinical characteristics in BC patients. (G, H) Kaplan–Meier survival curves for OS (G) and DFS (H) comparing the high and low expression of MTHFD2 in BC (GEPIA). (ns *p* ≥ 0.05; *p < 0.05; ***p < 0.001).

The clinical information of 433 BC patients in TCGA was collected and analysed. We found that the increased expression of MTHFD2 was significantly correlated with T stage (*p* < 0.001; Figure 2A), M stage (*p* = 0.028; Figure 2C), histologic grade (*p* < 0.001; Figure 2D), tumour subtype (*p* < 0.001; Figure 2E) and pathologic stage (*p* < 0.001; Figure 2F). Meanwhile, no significant associations were observed between MTHFD2 expression and other clinical features including N stage (*p* = 0.323; Figure 2B).

To explore the prognostic potential of MTHFD2 in BC, patients were divided into high‐expression and low‐expression groups according to the median expression of MTHFD2. We performed survival analysis based on the GEPIA. The Kaplan–Meier curve showed that high MTHFD2 expression was associated with poor overall survival (OS) (*p* = 0.0059; Figure 2G) and disease‐free survival (DFS) (*p* = 0.014; Figure 2H). Therefore, we got the conclusion that increased MTHFD2 expression predicts poor prognosis in BC.

### 3.3 MTHFD2 promoted cell growth of BC cells

**FIGURE 3:**
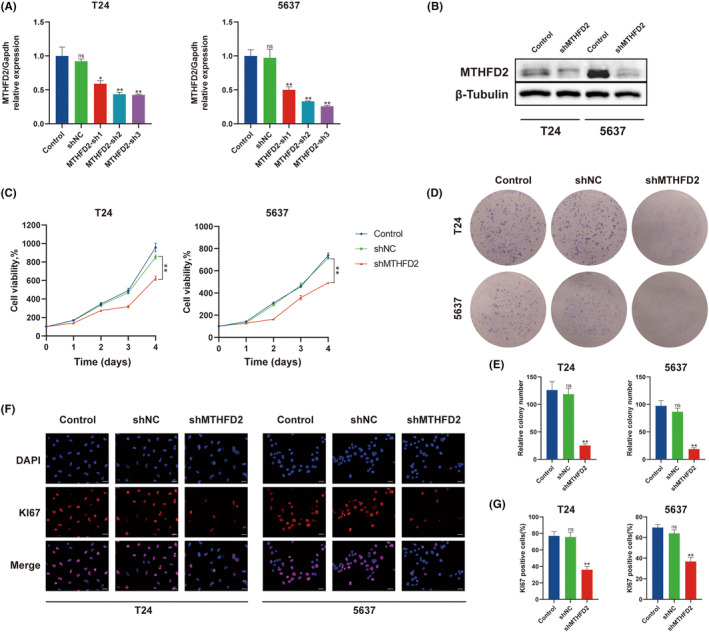
MTHFD2 enhanced proliferation of BC cells (A) Expression of MTHFD2 in T24 and 5637 cells analysed by RT‐qPCR after transfection with three shRNA mimics for MTHFD2. (B) WB analysis after transfection. (C) Cell viability was measured using the CCK‐8 for T24 and 5637 cells. (D, E) Colony formation assay for T24 and 5637 cells. (F, G) Representative immunofluorescence images of Ki67 staining in T24 and 5637 cells. (Scale bar represents 20 μm) (ns vs. Control *p* ≥ 0.05; * vs. shNC *p* < 0.05; ** vs. shNC *p* < 0.01).

To further explore the biological function of MTHFD2 in BC, we constructed MTHFD2 stable knockdown T24 and 5637 BC cell lines. Three shRNA mimics significantly decreased MTHFD2 expression as determined by RT‐qPCR (Figure 3A). MTHFD2‐sh3, which yielded the strongest reductions in MTHFD2 mRNA, was selected for further experimentation. The effect of MTHFD2‐sh3 knockdown was further verified by WB analysis (Figure 3B). Next, the results of CCK‐8 assay showed that the knockdown of MTHFD2 suppressed the proliferation ability of BC cells (Figure 3C), which were confirmed by colony formation assay (Figure 3D,E). Furthermore, we used immunofluorescence to measure the proliferation marker Ki67. The results showed that the knockdown of MTHFD2 inhibited Ki67 expression in BC cells (Figure 3F,G).

### 3.4 MTHFD2 promoted migration and invasion of BC cells

**FIGURE 4:**
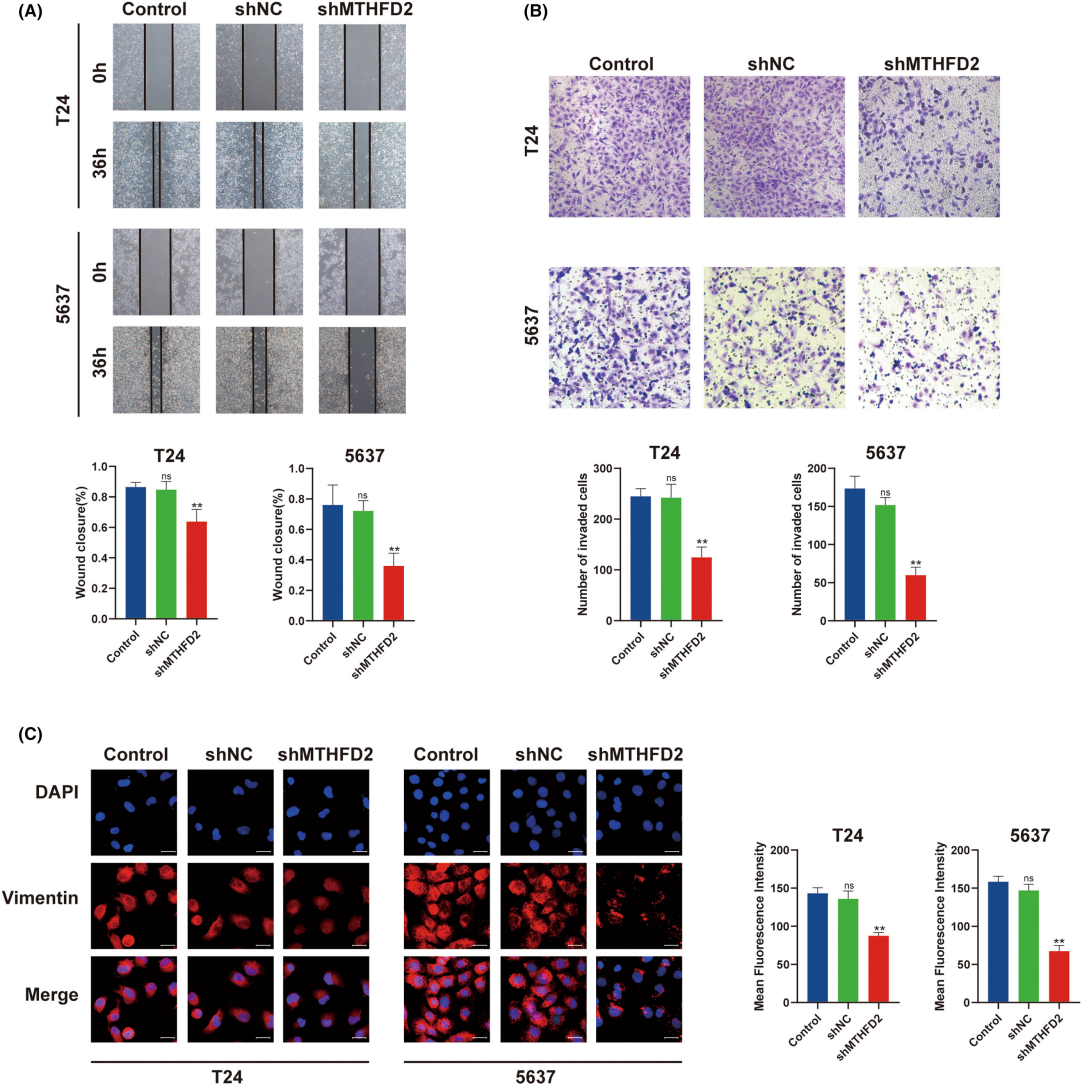
MTHFD2 enhanced migration and invasion of BC cells (A) Wound healing assay was performed to evaluate cell migratory ability for T24 and 5637 cells. (B) Transwell cell invasion assay was performed to evaluate cell invasive ability for T24 and 5637 cells. (C) Representative immunofluorescence images of vimentin staining in T24 and 5637 cells. (Scale bar represents 20 μm) (ns vs. Control *p* ≥ 0.05; ** vs. shNC *p* < 0.01).

We evaluated the effect of MTHFD2 on BC cell migration by wound healing assay. At 36 h after wound production, we found that wound healing was much slower in the MTHFD2 knockdown group compared to the negative controls (Figure 4A), suggesting that MTHFD2 could promote BC cell migration. To detect the effect of MTHFD2 on BC cell invasion, we used the transwell cell invasion assay. Compared with the shNC group, the number of invaded cells was significantly decreased in the MTHFD2 knockdown group (Figure 4B). Furthermore, immunofluorescence showed that the expression level of mesenchymal marker (vimentin) was downregulated after MTHFD2 knockdown (Figure 4C).

### 3.5 MTHFD2 inhibited apoptotic activity, maintained MMP and promoted cell cycle of BC cells

**FIGURE 5:**
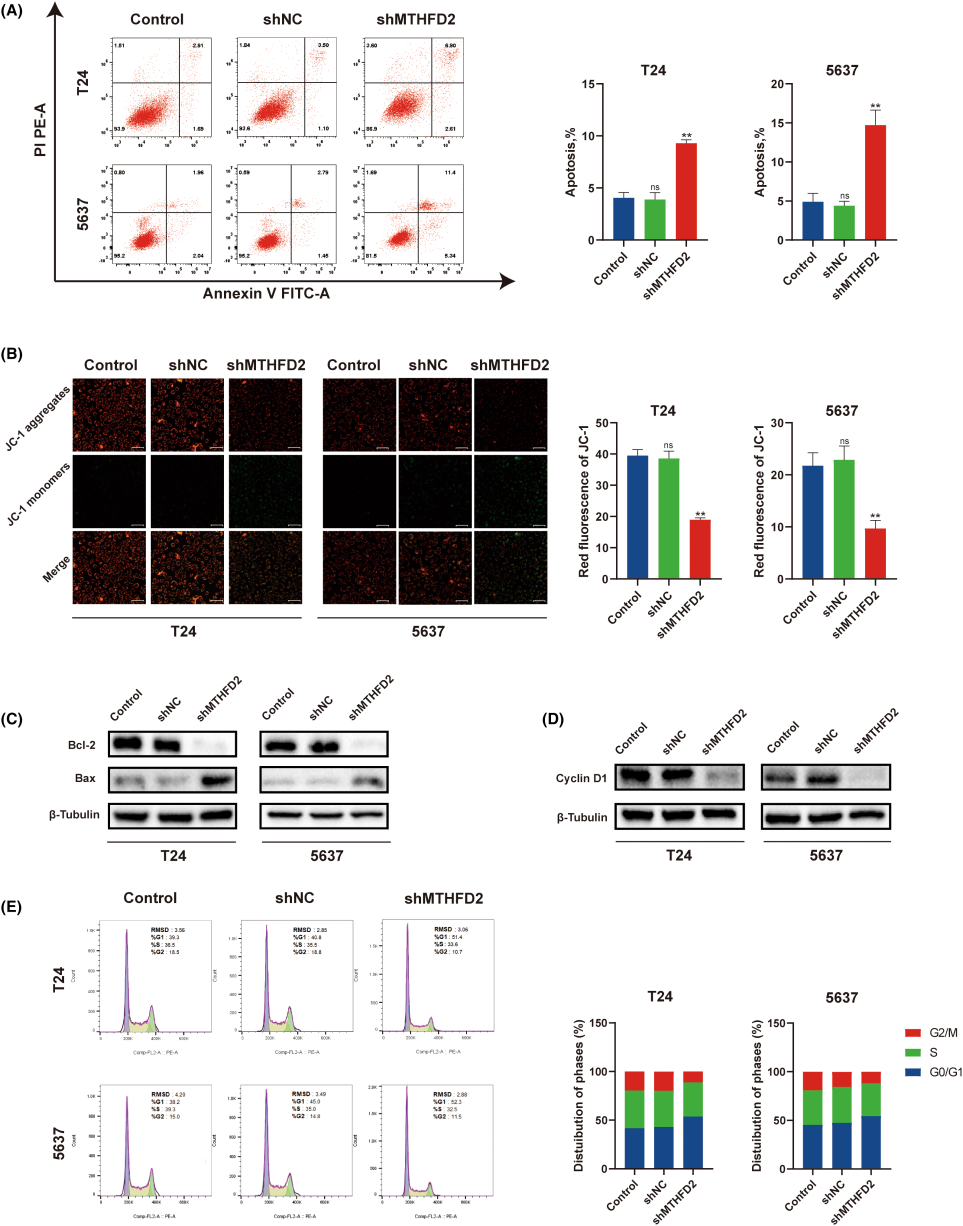
MTHFD2 inhibited apoptotic activity, maintained MMP, and promoted cell cycle of BC cells (A) Cell apoptosis was measured by flow cytometry for T24 and 5637 cells; (B) JC‐1 was observed as green monomers or as red aggregates. The micrographs were recorded under a fluorescent inverted microscope (OLYMPUS IX71) (scale bar represents 50 μm), and the red JC‐1 fluorescence intensity was quantified by ImageJ. (C, D) Protein expression levels of Bcl‐2, Bax, and cyclin D1. (E) Cell cycle analysis was measured by flow cytometry for T24 and 5637 cells. (ns vs. Control *p* ≥ 0.05; ** vs. shNC *p* < 0.01).

To investigate the role of MTHFD2 on apoptotic activity of BC cells, we performed flow cytometry with Annexin V and PI double staining in T24 and 5637 cells. The apoptosis rate of MTHFD2 knockdown cells was upregulated compared to the negative controls (Figure 5A). MMP is related to mitochondrial function, which is essential for cell survival. A dissipation in MMP is a sign of early apoptosis. To further explore the potential mechanism of MTHFD2 inhibiting apoptosis, we used JC‐1 dye to determine MMP in BC cells. Red JC‐1 aggregates indicate normal polarized mitochondria, whereas green JC‐1 monomers indicate loss of MMP. According to the results, the knockdown of MTHFD2 significantly decreased MMP in BC cells (Figure 5B). In addition, the expression of Bax was upregulated, whereas Bcl‐2 was downregulated in the shMTHFD2 group (Figure 5C).

To investigate whether the low proliferative capacity and high apoptotic activity of BC cells in the shMTHFD2 group were related to cell cycle arrest, we used flow cytometry to detect the cell cycle. We found an increase in the percentage of cells in the G0/G1 phase (T24, *p* < 0.01; 5637, *p* < 0.05) and a decrease in the G2/M phase (T24, *p* < 0.01; 5637, *p* < 0.05) in the shMTHFD2 group, indicating the cell cycle was blocked in G1 phase (Figure 5E). The expression of cyclin D1, which drives the cells from G1 to S phase, was downregulated in the shMTHFD2 group (Figure 5D).

### 3.6 MTHFD2 expression was correlated with immune infiltration levels and PD‐L1 expression

**FIGURE 6:**
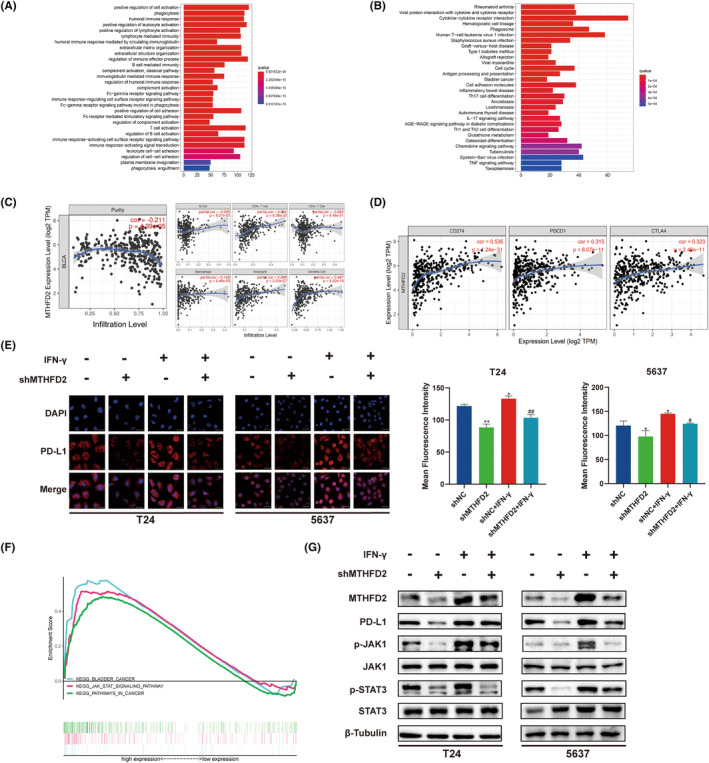
MTHFD2 expression was correlated with immune infiltration levels and PD‐L1 (A, B) GO terms and the KEGG pathways of MTHFD2 in TCGA database. (C) The correlation of MTHFD2 expression with diverse immune cells infiltration levels in BC from TIMER database. (D) The correlation between MTHFD2 expression and immune checkpoint inhibitor target genes, including CD274, PDCD1 and CTLA4. (E) PD‐L1 surface‐antigen detection using immunofluorescent staining. (Scale bar represents 20 μm) (F) GSEA results of MTHFD2 in TCGA database. (G) WB analysis of MTHFD2, PD‐L1, and JAK/STAT signalling pathway‐related proteins expression. (* vs. shNC *p* < 0.05; ** vs. shNC *p* < 0.01; # vs. shNC+ IFN‐γ *p* < 0.05; ## vs. shNC+ IFN‐γ *p* < 0.01).

So far, we have revealed the protumoural role of MTHFD2 in BC by bioinformatics methods and cell assays in vitro. To further explore the biological processes enriched by MTHFD2 in BC, we performed pathway enrichment analysis for Gene Ontology (GO) and Kyoto encyclopedia of genes and genomes (KEGG) (Figure 6A,B). The results showed that MTHFD2 was significantly associated with tumour cell cycle, invasion, migration and immune system regulation in BC. To verify whether MTHFD2 was correlated with immune infiltration levels, we assessed the correlation of MTHFD2 expression with tumour purity and diverse immune cells infiltration levels from TIMER database. The results showed that MTHFD2 expression was negatively correlated with tumour purity (*r* = −0.211, *p* = 4.39e−5) and positively correlated with the levels of infiltrating immune cells, like CD8 + T cells (*r* = 0.364, *p* = 6.38e−13), macrophages (*r* = 0.145, *p* = 5.49e−3), neutrophils (*r* = 0.269, *p* = 2.03e−07) and dendritic cells (*r* = 0.407, *p* = 5.62e−16) (Figure 6C). In addition, a strong positive correlation was observed between MTHFD2 expression and common immune checkpoint molecules, including CD274 (PD‐L1) (*r* = 0.535, *p* = 1.24e−31), PDCD1 (*r* = 0.315, *p* = 8.07e−11) and CTLA4 (*r* = 0.323, *p* = 2.40e−11) (Figure 6D).

To further investigate whether MTHFD2 could promote PD‐L1 expression in BC cells, we employed IFN‐γ to activate the expression of PD‐L1 and detected the PD‐L1 cell surface‐antigen expression by immunofluorescence staining. As expected, the expression of PD‐L1 decreased in the shMTHFD2 group compared to the negative control group, and increased after IFN‐γ treatment (Figure 6E). These results indicated that the knockdown of MTHFD2 suppressed basal PD‐L1 and IFN‐γ‐induced PD‐L1 expression in BC cells.

To explore the underlying signalling pathways between MTHFD2 and PD‐L1, we performed GSEA in the TCGA database. The results showed that gene sets related to the JAK/STAT signalling pathway were enriched in the MTHFD2 highly expressed group (Figure 6F). Furthermore, we found that the expression of MTHFD2, PD‐L1 and phosphorylation of JAK1 and STAT3 increased in the IFN‐γ‐treated BC cells and decreased after MTHFD2 knockdown (Figure 6G).

### 3.7 MTHFD2 promoted the expression of PD‐L1 via activation of JAK/STAT signalling pathway in BC

**FIGURE 7:**
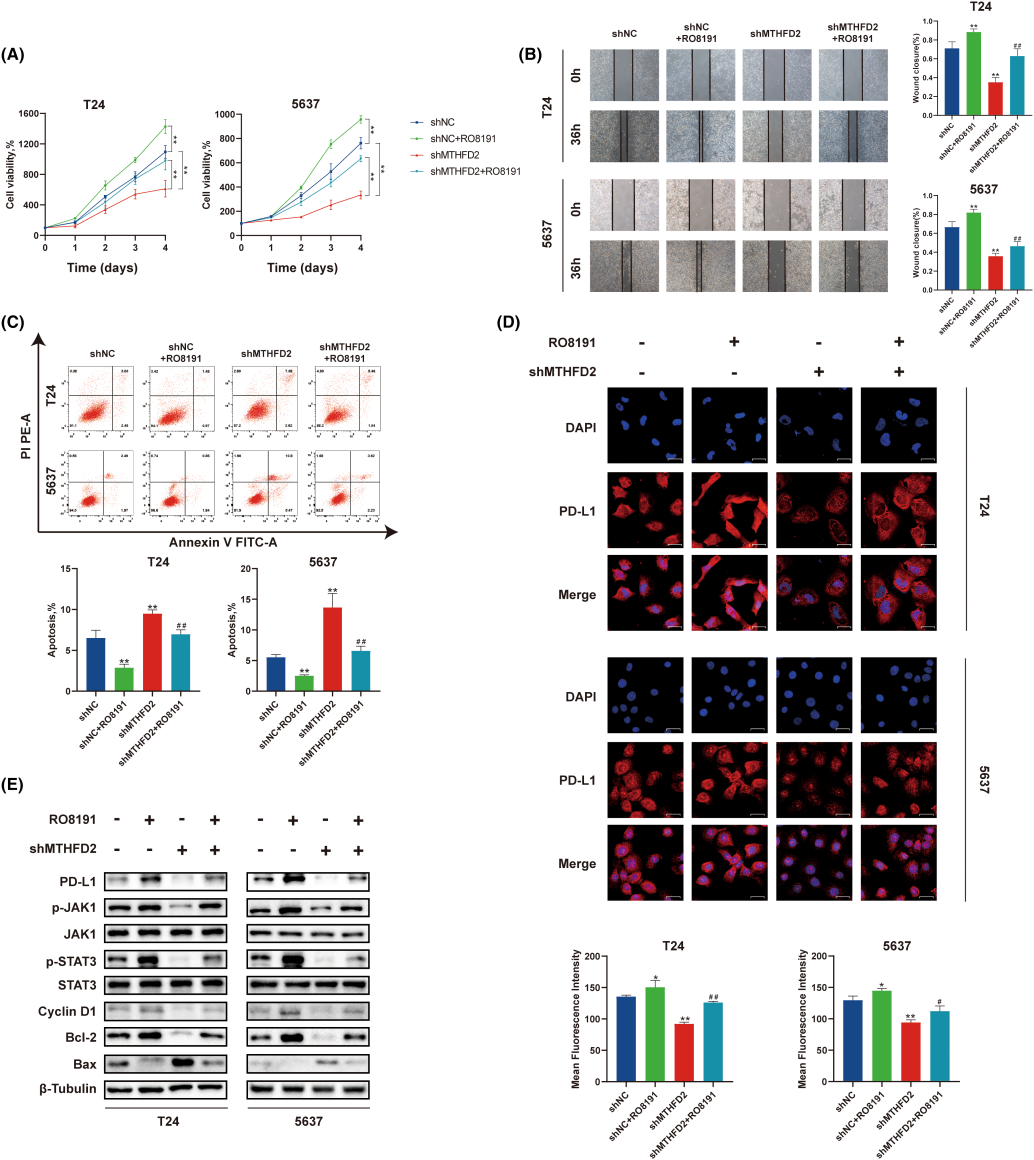
MTHFD2 promoted the expression of PD‐L1 via the JAK/STAT signalling pathway (A) Cell viability CCK‐8 assay. (B) Representative images of wound healing assay. (C) Cell apoptosis was measured by flow cytometry. (D) PD‐L1 surface‐antigen detection using immunofluorescent staining. (Scale bar represents 20 μm) (E) WB analysis of PD‐L1, cyclin D1, Bcl‐2, Bax and JAK/STAT signalling pathway‐related proteins expression. (* vs. shNC *p* < 0.05; ** vs. shNC *p* < 0.01; # vs. shMTHFD2 *p* < 0.05; ## vs. shMTHFD2 *p* < 0.01).

To further investigate whether MTHFD2 promotes the expression of PD‐L1 via the JAK/STAT signalling pathway, we employed the JAK/STAT pathway activator RO8191 to treat BC cells. We found that compared with shNC and shMTHFD2 groups, the relative cell activity and wound healing rate were significantly increased after the addition of RO8191, whereas the apoptosis rate was significantly decreased (Figure 7A–C). Using immunofluorescence staining, we found that the expression of PD‐L1 increased in the RO8191‐treated group (Figure 7D). Moreover, the expression levels of PD‐L1, cyclin D1, Bcl‐2, phosphorylation of JAK1 and STAT3 were increased in the RO8191‐treated group, while the levels of Bax were decreased (Figure 7E). Through immunohistochemical studies, we evaluated the expression of MTHFD2, PD‐L1 and phosphorylation of JAK1 and STAT3 in patient samples (Figure S4a). As expected, the expression of MTHFD2 correlated positively with the expression of PD‐L1 (*r* = 0.7434, *p* = 0.001), and phosphorylation of JAK1 (*r* = 0.7926, *p* < 0.001) and STAT3 (*r* = 0.8613, *p* < 0.001) in bladder cancer tissues (Figure S4b). These results suggested that MTHFD2 promoted the expression of PD‐L1 via activation of JAK/STAT signalling pathway.

### 3.8 MTHFD2 promoted BC growth in vivo

**FIGURE 8:**
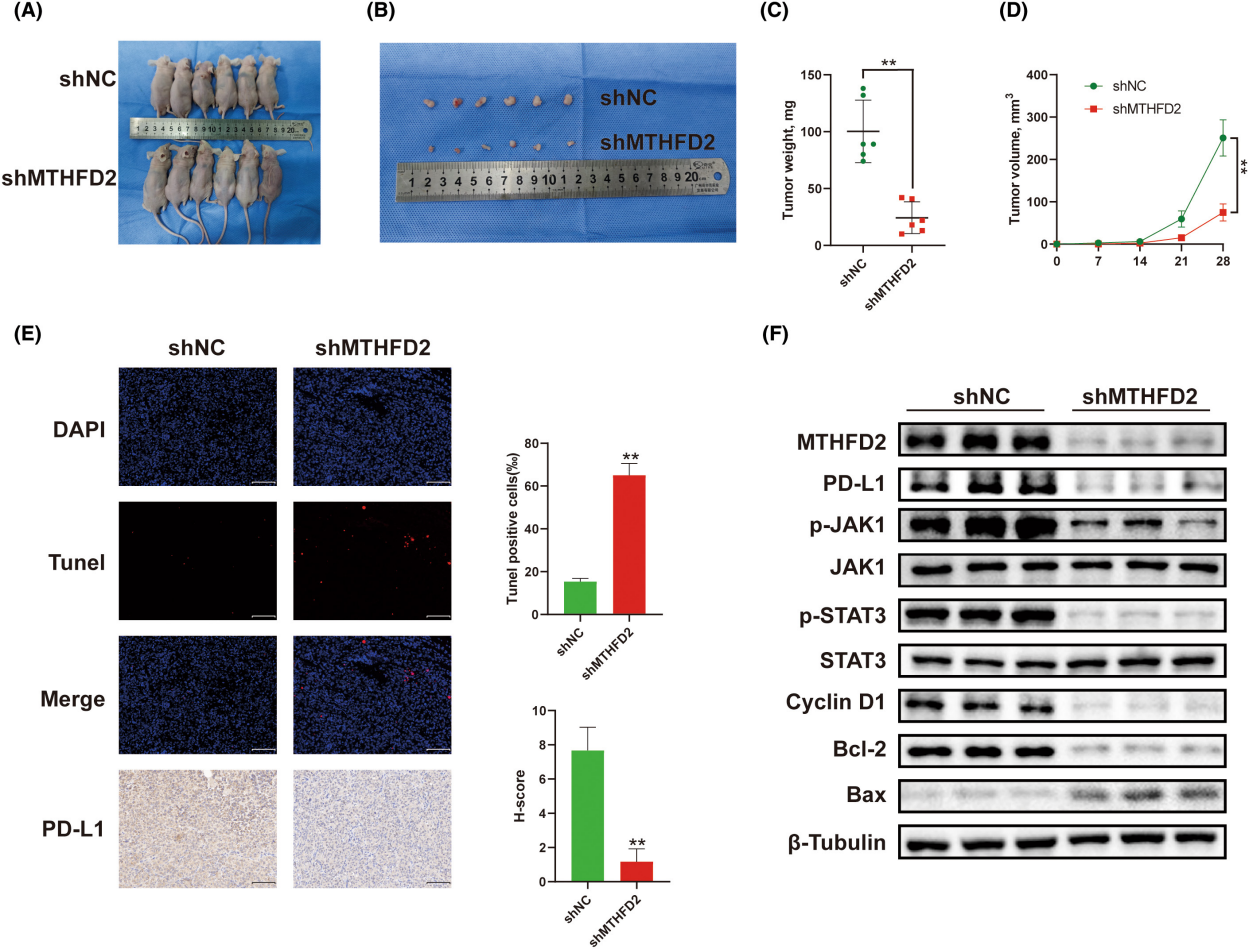
MTHFD2 promoted BC growth in vivo (A) Images of tumours in nude mice after subcutaneous injection with shNC or shMTHFD2 T24 cells. (B) Images of dissected tumours. (C, D) Tumour weight and volume. (E) Representative images of TUNEL staining and immunohistochemistry staining for PD‐L1 in different groups. (Scale bar represents 100 μm) (F) WB analysis of MTHFD2, PD‐L1, cyclin D1, Bcl‐2, Bax and JAK/STAT signalling pathway‐related proteins expression in different groups. (** vs. shNC *p* < 0.01).

To demonstrate the function of MTHFD2 in vivo, we established a T24 xenograft tumour model in nude mice. Nude mice were subcutaneously injected with shNC or shMTHFD2 T24 cells. As the results showed, the growth of tumour cell xenografts in the shMTHFD2 group was suppressed compared to the shNC group (Figure 8A–D). In addition, TUNEL staining results showed a significant increase in TUNEL‐positive cells in the shMTHFD2 group. Immunohistochemistry showed that compared to the shNC group, the PD‐L1 expression of tumours in the shMTHFD2 group decreased (Figure 8E). Furthermore, the lysates of pooled tumour tissues in nude mice were subjected to WB analysis. Similar to the previous results, the expression levels of MTHFD2, PD‐L1, cyclin D1, Bcl‐2 and phosphorylation of JAK1 and STAT3 were decreased in the shMTHFD2 group, while the levels of Bax were increased (Figure 8F).

## 4 DISCUSSION

BC is one of the most frequently diagnosed cancers worldwide and has high incidence and mortality rates.^18^, ^19^ Approximately 70% of patients with BC are diagnosed with non‐muscle‐invasive bladder cancer (NMIBC),^20^ which has a relatively good prognosis. However, half of NMIBC cases are likely to recur, and 20–30% of cases progress to secondary MIBC.^21^ MIBC has a poor prognosis, with a 5‐year survival rate of 60% for patients with localized disease and <10% for those with distant metastases.^22^ Despite recent advances in surgical techniques and drug therapy, the overall prognosis of BC has remained the same over the last three decades.^23^ Therefore, it is critical to explore the key molecular interactions involved in BC progression to provide new treatment options.

MTHFD2, a folate‐coupled mitochondrial metabolic enzyme,^24^ is upregulated in many types of rapidly proliferating cancers such as breast cancer and colorectal cancer.^5^ High expression levels of MTHFD2 are associated with poor survival rates in many cancer types, such as colorectal cancer,^25^ lung adenocarcinoma,^26^ pancreatic cancer^27^ and ovarian cancer.^28^ Previous studies have shown that the inhibition of MTHFD2 expression suppresses malignant phenotypes in various cancers and that the effects of MTHFD2 depletion vary among different types of cancer cells.^29^ However, the biofunction of MHFD2 in BC has not yet been fully investigated. In the present study, we first analysed various databases and found that MTFHD2 was significantly upregulated in multiple types of cancers, including BC. We further demonstrated that MTHFD2 was highly expressed in BC cell lines and tissue samples via WB analysis, quantitative reverse transcription polymerase chain reaction and immunohistochemistry. Next, we found that an increased MTHFD2 expression was significantly correlated with clinical features and a poor prognosis in BC. Subsequently, we used T24 and 5637 BC cell lines to explore the cellular functions of MTHFD2 in vitro. Furthermore, a T24 xenograft tumour model was established in nude mice to demonstrate the functions of MTHFD2 in vivo. Our results showed that MTHFD2 promoted the proliferation, migration, invasion, and tumourigenicity and inhibited the apoptosis of BC cells. In agreement with previous studies, our results suggest that MTHFD2 promotes malignant phenotypes and is a potential novel biomarker for BC.

As a vital part of cancer treatment, immunotherapy has demonstrated survival benefits and durable clinical responses in patients with cancer.^30^ Currently, ICI are being used in the treatment of various cancer types, including BC, and their use is expected to increase substantially in the coming years.^31^ PD‐L1 is a crucial immune checkpoint, and anti‐PD‐L1 antibodies have been approved for the treatment of metastatic BC.^32^ For PD‐L1‐positive patients with BC, anti‐PD‐L1 therapy has been shown to be significantly effective.^2^ In addition, novel combination strategies involving anti‐PD‐L1 therapy and targeted therapy have been developed and pursued to extend the benefits of ICI.^33^ Therefore, it is critical to understand how the expression of PD‐L1 is regulated in BC. A recent study showed that MTHFD2 promotes tumour immune evasion by upregulating PD‐L1.^6^ However, this finding has not yet been demonstrated in patients with BC. We analysed the TIMER database and performed immunofluorescence staining. As expected, MTHFD2 expression was correlated with immune infiltration and PD‐L1 expression in BC. To further explore the underlying mechanisms, we performed gene set enrichment analysis and found that the JAK/STAT signalling pathway was related to MTHFD2. Activation of the JAK/STAT signalling pathway has been reported to contribute to tumour cell immune escape, proliferation, angiogenesis and survival.^34^, ^35^ Moreover, previous studies have demonstrated that IFN‐γ, as an essential effector molecule for immune responses, can induce tumour progression and stimulate the expression of PD‐L1 via the JAK/STAT3 signalling pathway.^36^, ^37^ Therefore, in the present study, IFN‐γ was used to activate PD‐L1 expression. WB analysis showed that PD‐L1 and MTHFD2 were upregulated and the JAK/STAT signalling pathway was activated in IFN‐γ‐treated BC cells. However, knockdown of MTHFD2 suppressed the effects of IFN‐γ on BC cells. Subsequently, we used the JAK/STAT pathway activator RO8191 to further demonstrate the relationship between MTHFD2 expression and the JAK/STAT signalling pathway. As expected, addition of RO8191 partially reduced the effect of MTHFD2 knockdown on proliferation, migration and other biological functions in BC cells. These results further confirmed that MTHFD2 upregulates PD‐L1 by activating the JAK/STAT signalling pathway, which suggests that combining MTHFD2 inhibitors with PD‐L1 inhibitors may be a novel immunotherapy for BC and warrants further investigation. However, it is common for molecules to affect gene expression and regulate tumourigenesis through multiple pathways. It has been reported that MTHFD2 promotes cMYC O‐GlcNAcylation through uridine‐related metabolites, resulting in increased cMYC stability and PD‐L1 transcription.^6^ Moreover, MTHFD2 could encourage cell cycle progression by activating CDK2 and sequentially affecting E2F1 activation.^38^ In lung adenocarcinoma, MTHFD2 promoted cell growth and metastasis via AKT/GSK‐3β/β‐catenin signalling.^39^ Therefore, whether MTHFD2 has other downstream molecules and pathways in BC needs to be studied.

## 5 CONCLUSIONS

This study suggests that MTHFD2 is highly expressed and promotes malignant phenotypes in patients with BC. The expression of MTHFD2 is correlated with immune infiltration levels and promotes PD‐L1 expression in BC via the JAK/STAT signalling pathway. Thus, MTHFD2 is a novel potential biomarker for disease prognosis and targeted therapy.

## AUTHOR CONTRIBUTIONS

**Linzhi Li:** Conceptualization (equal); data curation (lead); formal analysis (lead); resources (lead); validation (equal); writing – original draft (equal); writing – review and editing (equal). **Yunlong Zhang:** Conceptualization (equal); data curation (equal); formal analysis (supporting); resources (equal); validation (equal); visualization (equal). **Weimin Hu:** Data curation (equal); resources (equal). **Fan Zou:** Data curation (equal); resources (equal). **Jin‐Zhuo Ning:** Investigation (equal); project administration (equal); supervision (equal). **Ting Rao:** Funding acquisition (equal); investigation (equal); supervision (equal). **Yuan Ruan:** Funding acquisition (equal); investigation (equal); supervision (equal). **Weimin Yu:** Methodology (equal); project administration (equal). **Fan Cheng:** Funding acquisition (lead); project administration (lead).

## FUNDING INFORMATION

This work was funded by the National Natural Science Foundation of China [No. 82170775; No. 82100806], Science and Technology Major Project of Hubei Province [No. 2020BCB017; No. 2019AEA170] and Open Fund of Hubei Key Laboratory [2021KFY039].

## CONFLICT OF INTEREST STATEMENT

The authors declare that there is no conflict of interest regarding the publication of this paper.

## Supporting information


Figures S1

## Data Availability

The raw data used to support the findings of this study are available from the authors, without undue reservation.
