# Supplementary material for: MTHFD2 promotes PD‐L1 expression via activation of the JAK/STAT signalling pathway in bladder cancer
Source: J Cell Mol Med. 2023 Jul 21;27(19):2922–36. doi: 10.1111/jcmm.17863 (PMC10538262; doi:10.1111/jcmm.17863)
Supplement: Supplementary file 1 — Figures S1 [file JCMM-27-2922-s001.docx]

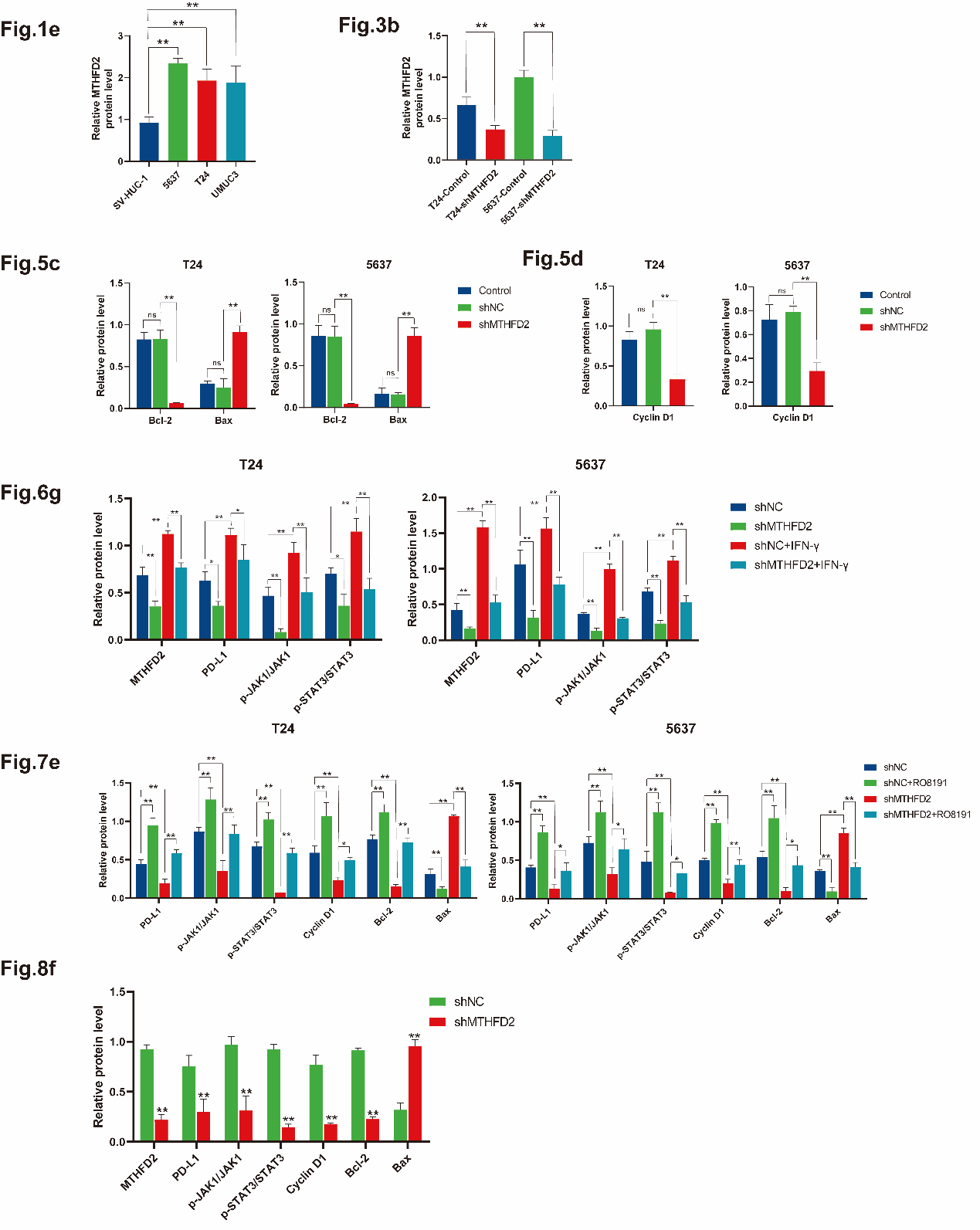


**Figure S1:** The statistical graphs about WB in all figures. (ns p ≥ 0.05; * p < 0.05; ** p < 0.01)


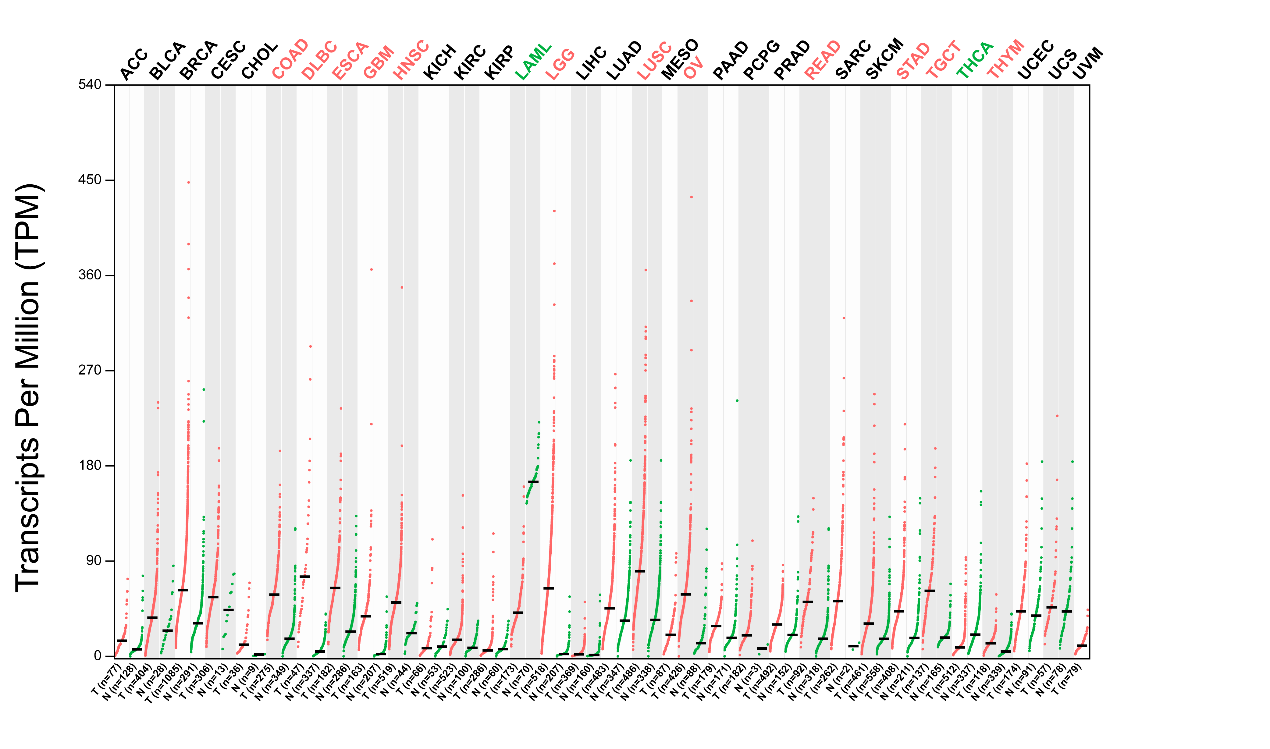


**Figure S2:** Differential expression of MTHFD2 in various types of cancer analyzed by GEPIA.


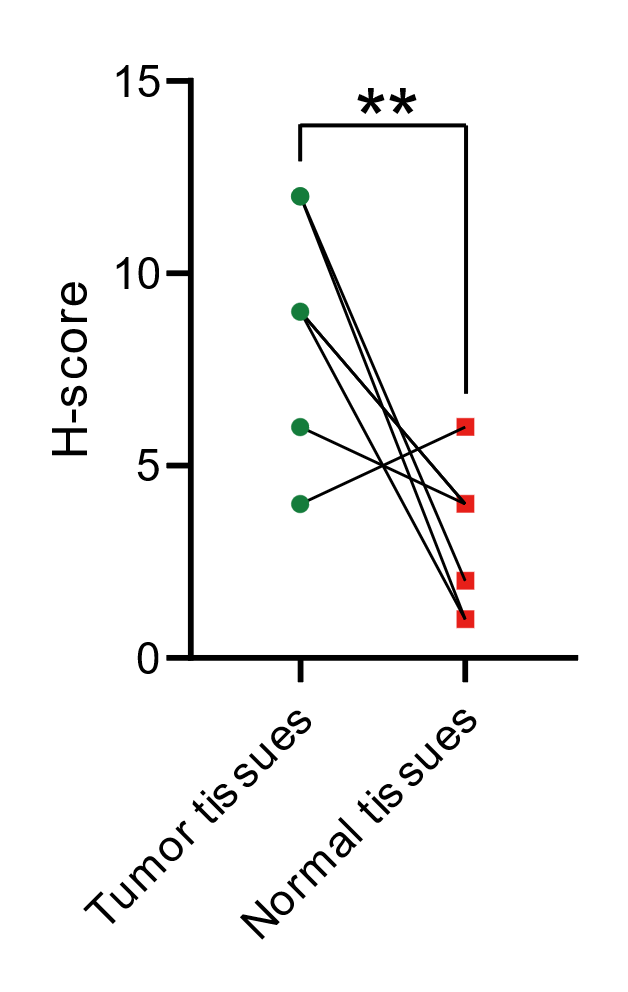


**Figure S3:** Quantitative analysis of MTHFD2 expression levels in patient samples. H-score of the clinical tumor and adjacent normal tissues of 8 BC patients. (** p < 0.01)


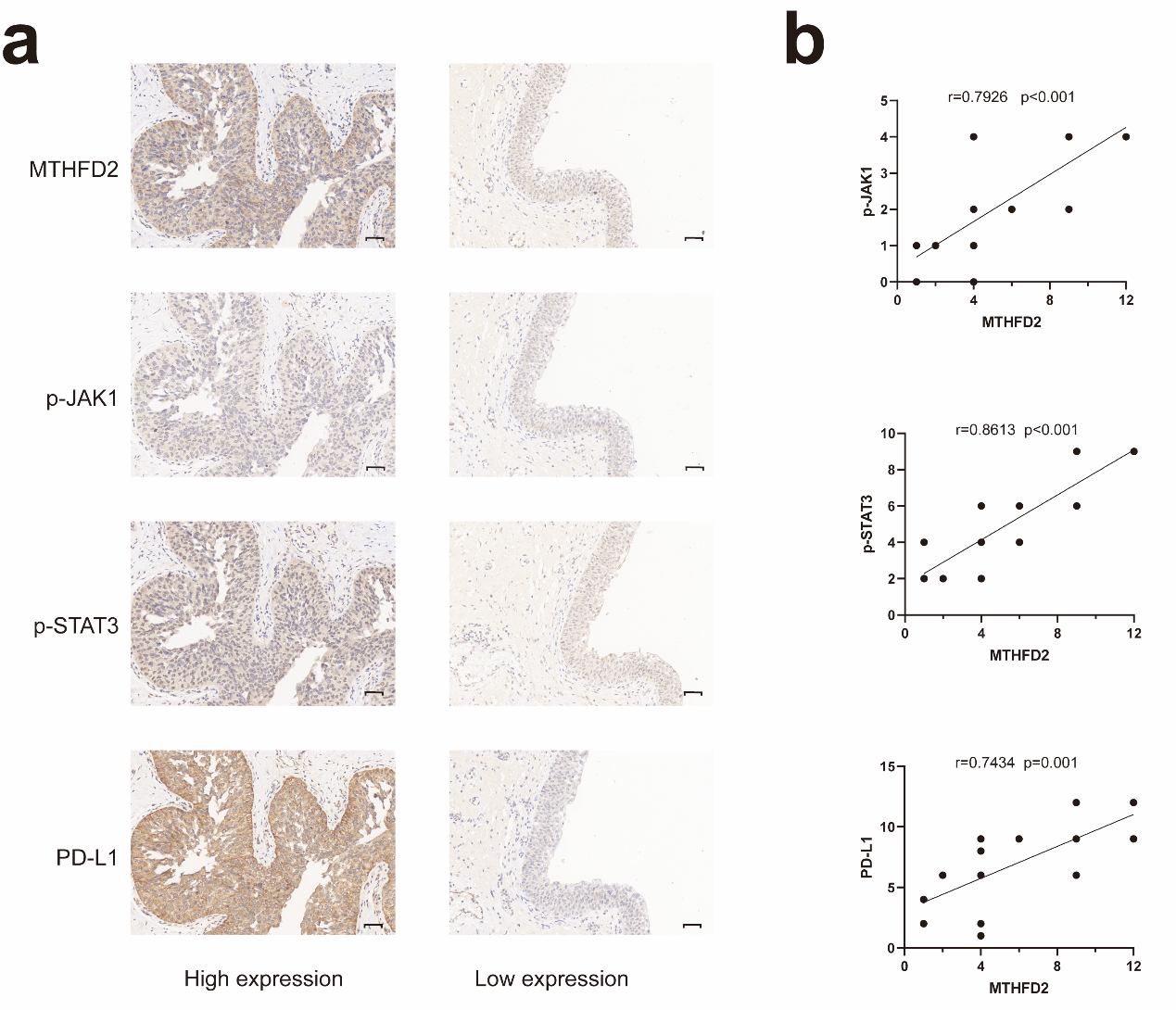


**Figure S4:** The correlation of MTHFD2 and JAK/STAT3 pathway/PD-L1 in patient samples (a) Representative immunostaining pictures of MTHFD2, p-JAK1, p-STAT3 and PD-L1 in patient samples. (Scale bar represents 100 μm) (b) Linear regression analysis of MTHFD2 and p-JAK1, p-STAT3 and PD-L1 H-score of immunostaining in patient samples.
